# HDPE/Chitosan Composites Modified with PE-g-MA. Thermal, Morphological and Antibacterial Analysis

**DOI:** 10.3390/polym11101559

**Published:** 2019-09-25

**Authors:** Poliana S. Lima, Rossanna Trocolli, Renate M. R. Wellen, Luis Rojo, Miguel A. Lopez-Manchado, Marcus V. L. Fook, Suédina M. L. Silva

**Affiliations:** 1 Department of Materials Engineering, Federal University of Campina Grande, Campina Grande, PB 58429-900, Brazil; polianassilva@yahoo.com.br (P.S.L.); rossannatrocolli@gmail.com (R.T.); marcus.liafook@certbio.ufcg.edu.br (M.V.L.F.); 2 Department of Materials Engineering, Federal University of Paraíba, João Pessoa, PB 58051-900, Brazil; wellen.renate@gmail.com; 3 Institute of Polymer Science and Technology (ICTP- CSIC), Juan de la Cierva 3, 28006 Madrid, Spain; rojodelolmo@ictp.csic.es (L.R.); lmanchado@ictp.csic.es (M.A.L.-M.)

**Keywords:** HDPE, PE-g-MA, chitosan, bacteriostatic effect, thermal and morphological properties

## Abstract

In this work, composites of high density polyethylene (HDPE) with chitosan were prepared by melt compounding in a laboratory internal mixer. Maleic anhydride grafted HDPE (PE-g-MA) in a concentration up to 25 phr was used as a compatibilizer to enhance the dispersing effect of chitosan in the HDPE matrix. The degree of crystallinity was investigated by X-ray diffraction (XRD) and the thermal properties were analyzed by differential scanning calorimetry (DSC) and thermogravimetry (TG). The morphology was investigated by optical microscopy (OM) and scanning electron microscopy (SEM). The integrity of composites was evaluated by mechanical properties and antibacterial properties were assessed against *Escherichia coli* (DH5a). Neither crystallinity nor HDPE’s melting parameters changed upon addition of chitosan and PE-g-MA. Chitosan aggregates were observed, which were dispersed upon addition of PE-g-MA, which also provided improved mechanical performance. Chitosan significantly improved the bacteriostatic effect of HDPE compounds preventing bacteria to colonize thus reducing the number of viable colony-forming units (CFU). This study revealed that HDPE/chitosan composites could be obtained by melt compounding, at lower cost and additionally having antibacterial properties, which might provide a new formulation option for developing antimicrobial film for food packaging.

## 1. Introduction

Polymers have supplied most of the common materials in modern society because they present several desired features like versatility, lightness, easy processability, softness and low cost being suitable for multiple uses such us packaging, automotive, medical devices fabrication, construction and electro-electronics industries among many others [1,2,3].

Fossil fuel used for both energy production and plastic manufacturing has finite availability. Approximately 6–8% of the global production of fossil fuel goes into the synthesis of plastic materials such as polyolefin, polystyrenes, polyesters or polyamides. However, the environmental concerns about the lack on biodegradability have led to an increased interest in the development of green plastics and composite materials derived from renewable natural resources with distinct or superior physical and chemical properties [4,5,6].

Polythene shares about 64% among the synthetic plastics waste produced, and it is considered as the most commonly found solid waste that has been recognized as a major threat to marine life [7,8]. Although their complete replacement for eco-friendly polymers is just far from current development, at least for specific applications, the use of PE based composites in which bioplastics and other fillers that enhance its biodegradability and avoid leaching harmful products to the environment, should be encouraged [9,10,11,12].

In contrary, chitosan is a natural polysaccharide obtained from the deacetylation of chitin, one of the world’s most plentiful, renewable organic resources; it is completely biodegradable and can be manufactured in the form of films, strips and gels. Chitosan has important biological (biocompatible), physiological and pharmacological properties, being a very functional molecule, performing healing activity, immunostimulant, antitumor and antibacterial activities. Its production is relatively cheap, as an eco-friendly material it is safe for humans and for the environment [13,14,15].

Polyethylene blended with chitosan prepared by melt processing has been reported in numerous publications and has been considered one of the best approaches for preparing materials partially or totally biodegradable because of their high yield and greater control of the final material’s characteristics without atmospheric pollution, in comparison with the solvent evaporation methods [16,17,18,19,20,21,22,23,24,25,26,27,28,29,30,31,32,33,34]. Nevertheless, more effort is still required to develop a HDPE blend containing chitosan with improved rheological, thermal, mechanical, morphological and antibacterial.

In light of this, in this work we present the preparation of composite materials in which different proportions of biodegradable chitosan have been introduced into the PE matrix, previously modified with grafted maleic anhydride as a compatibilizer, and studied its compatibility, physicochemical properties and mechanical performance. In addition, some beneficial features such as antibacterial properties have been evaluated to the final composites.

## 2. Materials and Methods

### 2.1. Materials

High density polyethylene (HDPE), injection molding grade JV060U, was supplied by Braskem (São Paulo, Brazil), it is a 1-butene copolymer of narrow molecular weight. It contains antioxidants and UV stabilizer additives, with a density of 0.957 g/cm^3^ (ASTM D 792) and a melt flow index (MFI) of 7 g/10 min (ASTM D1238, 190 °C/2.16 kg) [34]. The density of HDPE at the processing temperature (about 190 °C) was estimated in 0.76 g/cm^3^ [35]. Medium molecular weight chitosan from shrimp shells, Mv = 114 kDa determined by viscometry [36] and degree of acetylation (DA) 8%, determined by the infrared spectroscopy method [37], was supplied by Polymar-Fortaleza/CE-Brazil. The true density of chitosan was estimated as 1.48 g/cm^3^ (at processing conditions) [38,39]. Maleated polyethylene (PE-*g*-MA), Polybond 3009, supplied by Chemtura (São Paulo, Brazil), is a grafted linear polyethylene with 1% maleic anhydride content and MFI of 6 g/10 min (ASTM D1238, 190 °C/2.16 kg). It was used as a compatibilizer in HDPE/chitosan compounds.

### 2.2. Preparation of HDPE/Chitosan Composites

HDPE/chitosan composites were prepared in a Haake Rheomix 3000 laboratory internal mixer (Thermo Fisher Scientific, Waltham, MA, USA) fitted with high intensity rotors (roller type) operated at 60 rpm with the chamber wall maintained at 180 °C. HDPE (as received) without and with PE-*g*-MA compatibilizer (dried under vacuum for 24 h at 80 °C) was first loaded. After 6 min, chitosan (dried under the same conditions) was added without interrupting the process. Mixing continued for another 4 min. HDPE/chitosan composites were removed, dried at room temperature, ground and stored in sealed containers. Sample compositions prepared are summarized in Table 1. PE-*g*-MA and chitosan were coded as C and Q, respectively. A detailed rheological study of compounds prepared in this work was reported in our previous study [34].

Injected specimens for mechanical tests were produced according to ASTM D638 and ASTM D256 using a FLUIDMEC H3040, the injection temperature was 140 °C with a mold at 20 °C.

### 2.3. Characterization of the Samples

#### 2.3.1. X-ray Diffraction (XRD)

XRD experiments carried out in a Shimadzu XRD-7000 (Shimadzu, Kyoto, Japan) in the region of 2–30° (2θ), with λ_CuKα_ = 0.1542 nm radiation, tension 40 kV, current 30 mA and scan rate 2°/min. Powder chitosan was used whereas HDPE composites were tested as compressed films with thickness ranging from 0.3 to 0.7 mm. From XRD diffractograms the crystallinity of specimens was evaluated following Ruland^26^ methodology, according to Equation (1):

(1) $$\%C=100\left(\frac{Ic}{Ic+K*Ia}\right)$$

where: %*C* is the crystallinity fraction; Ic is the area of diffraction peaks; Ia is the area of amorphous halo and *K* is a proportionality constant (K = 1.0).

#### 2.3.2. Differential Scanning Calorimetry (DSC)

Differential scanning calorimetry (DSC) analyses were acquired in a TA Instrument DSC Q20 (TA Instruments, New Castle, PA, USA) under a nitrogen flow of 50 mL/min, using aluminum pans. Samples tests weighting between 6 and 10 mg were used. The thermal cycles were composed of three stages: (1) Heating from 20 °C to 160 °C, keeping at this temperature for 3 min (isothermal process); (2) cooling from 160 °C to 20 °C and (3) re-heating from 20 °C to 160 °C. During the thermal cycles a heating/cooling rate of 10 °C/min was applied.

#### 2.3.3. Thermogravimetry (TG)

Thermogravimetry analyses (TG) were performed in a Shimadzu TGA S1HA (Shimadzu, Kyoto, Japan). Samples with approximately 5 mg were heated in an alumina pan from 30 °C to 800 °C using a heating rate of 10 °C/min, under a nitrogen flow of 50 mL/min. Differential thermogravimetry (DTG) scans were also computed.

#### 2.3.4. Optical Microscopy (OM)

Optical microscopy images of compressed HDPE films were captured in a Leica Microsystems 750, images from the edge and center were analyzed.

#### 2.3.5. Scanning Electron Microscopy (SEM)

SEM images were obtained in a SSX 550 Superscan—SHIMADZU equipment (Shimadzu, Kyoto, Japan) operating at 15 kV, under high vacuum. Films were fractured in liquid nitrogen and their surfaces were gold coated using a Sanyu Electron SC-701 sputter, operating with a 10 mA current for a period of 4 min, it was done in order to avoid negative charge accumulation.

#### 2.3.6. Mechanical Properties

Mechanical properties in tension were measured according to ASTM D638 using specimens type IV. Tests were performed in a LLOYD LR-10k testing machine operating at 10 mm/min with cell charge of 10 kN. Elastic modulus, tensile strength and elongation at break results were automatically obtained from the equipment software. Mechanical tests were conducted in room temperature (~23 °C) and reported results are an average of six experiments.

Impact tests carried out in a CEAST Resil-5.5 impact machine operating with a 2.75 J pendulum and frictional energy of 0.015 J, according to ASTM D256. Notches with 1.5 mm were made in CEAST NOTSCHVIS according to the Izod configuration. Impact tests were conducted in room temperature (~23 °C) and presented results were an average of six experiments.

#### 2.3.7. Antibacterial Properties

Antibacterial properties of HDPE, HDPE/C10/Q10 and HDPE/C20/Q20 composites were assessed against *Escherichia coli* (DH5α). Stock bacteria storage at −80 °C in 15% of glycerol was transferred to a lysogeny broth (LB) agar plate and incubated for 22 h at 37 °C. Afterwards, a single colony was inoculated into 25 mL of the LB medium and incubated for 12 h at 37 °C. Bacterial concentration was adjusted with a sterile phosphate buffer saline (PBS) to an OD600 equivalent to 108 colony-forming units (CFU)/mL.

Environmental scanning electron microscopy (SEM) was used to evaluate the ability of bacteria to form biofilms and colony-forming units (CFU) counting and expressed as relative cell viability (RCV%).

Test disks of 14 mm diameter were placed into a separate well of a 24-well plate with the test surface facing up. The plate was introduced in a plastic container wet filter paper beneath to maintain a relative humidity of 90%. Of the test inoculums (10^5^ CFU·cm^−2^) 100 μL was prepared in 1/500 diluted NB were placed onto the substrates and incubated at 37 °C for 24 h. Afterwards, samples were washed with 1 mL of PBS (pH 7.2) and the numbers of CFU recovered from each sample disks were determined by standardized plate counting agar techniques. RCV was determined by the number of CFU (N)/cm^2^ referred to the uncoated control RCV (%) = N/Ncontrol × 100.

For SEM observation, disks were inoculated as described and incubated at 37 °C for 24 h. Afterwards each disc was washed three times with distilled sterile water and fixed with 2.5% glutaraldehyde for 2 h at room temperature. The dried samples were mounted on aluminum stumps and sputter-coated with chromium before examination under an SEM apparatus (Philips XL 30) at an accelerating voltage of 15 kV.

All data were expressed as the mean ± standard deviation (*n* = 3) and statistical analyses were performed using ANOVA, employing Origin Software version 8.6 (OriginLab, Northampton, MA, USA). Differences with *p* < 0.05 were considered statistically significant.

## 3. Results and Discussion

**Figure 1:**
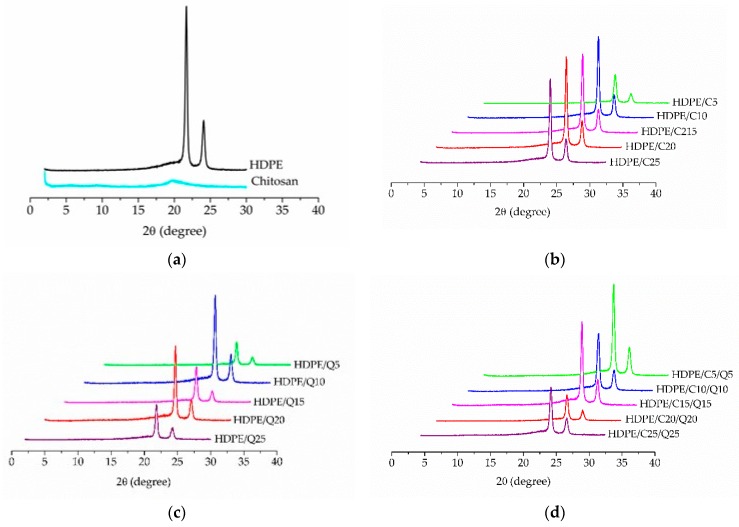
XRD diffractograms: (**a**) high density polyethylene (HDPE) and chitosan; (**b**) HDPE/C compounds; (**c**) HDPE/Q compounds and (**d**) HDPE/C/Q compounds.

X-ray diffractograms of samples investigated in this work are shown in Figure 1, in (a) the HDPE diffractogram presents crystalline peaks at 2θ 21.6° and 24.2°, which is evidence of the HDPE orthorhombic crystalline structure [40,41]; for chitosan a peak is observed at 2θ 20.1° confirming its crystallinity [42].

Figure 1b–d shows diffractograms of HDPE/C, HDPE/Q and HDPE/C/Q compounds, respectively, they illustrate the characteristic peaks of HDPE. It is verified that the HDPE peak areas did not change upon the addition of chitosan and a compatibilizer, where its crystalline character was clearly displayed.

The degree of crystallinity for neat HDPE, PE-g-MA, chitosan and HDPE/C, HDPE/Q and HDPE/C/Q compounds was measured from X-ray diffractograms using Equation (1), and the results are displayed in Supplementary Material Table S1. As can be verified, the addition of PE-g-MA and chitosan lightly influenced the crystallinity of HDPE, these results were consistent with those presented by DSC experiments.

**Figure 2:**
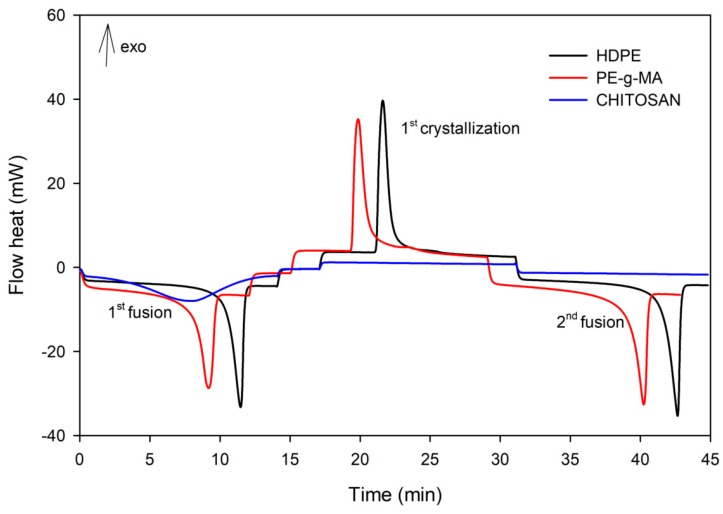
Differential scanning calorimetry (DSC) scans of HDPE, maleic anhydride grafted HDPE (PE-*g*-MA) and chitosan.

Figure 2 presents DSC scans of HDPE, PE-g-MA and chitosan heat cycled by the thermal program as described in the methodology. DSC scans of HDPE and PE-g-MA present endothermic peaks during the first and second heating due to the melting, and an exothermic peak during the cooling due to melt crystallization. DSC of chitosan did not present peaks related to phase changing, however between 70 and 120 °C, an endothermic peak was observed probably related to the water evaporation [40].

**Figure 3:**
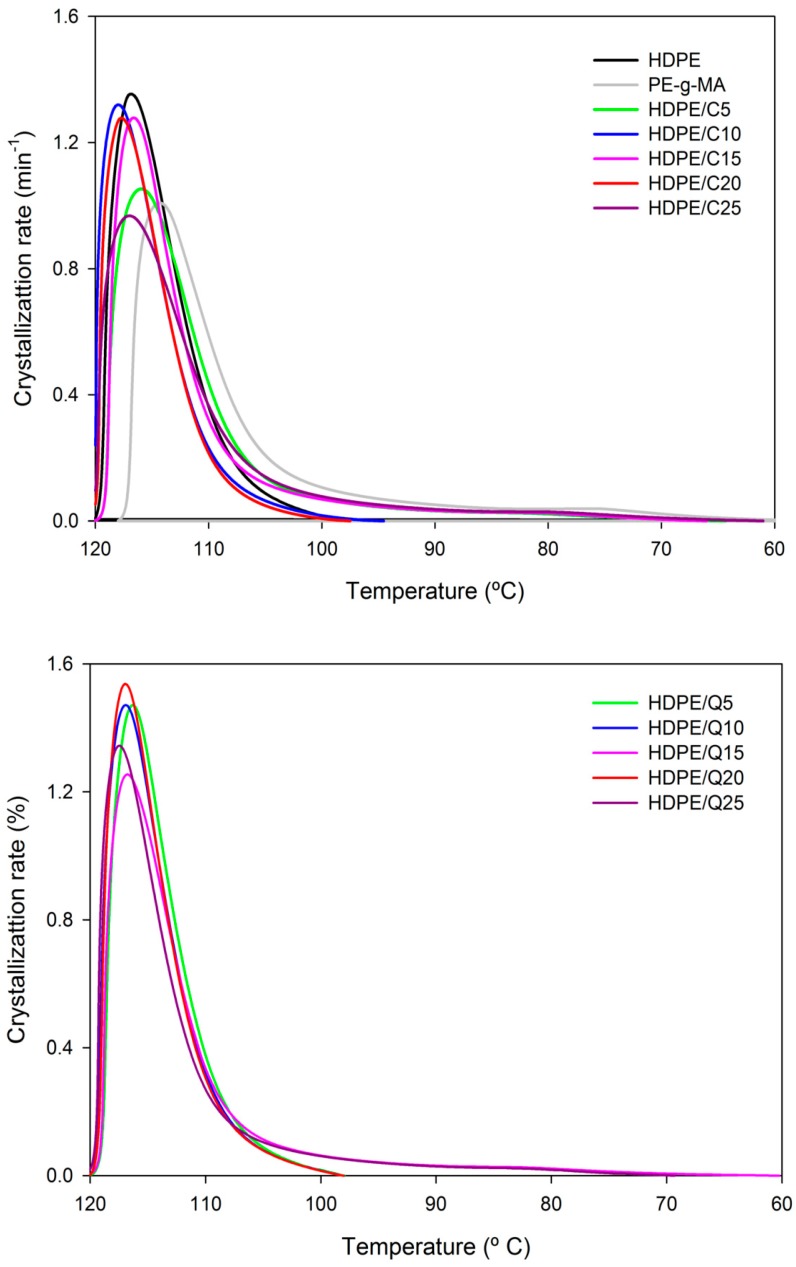

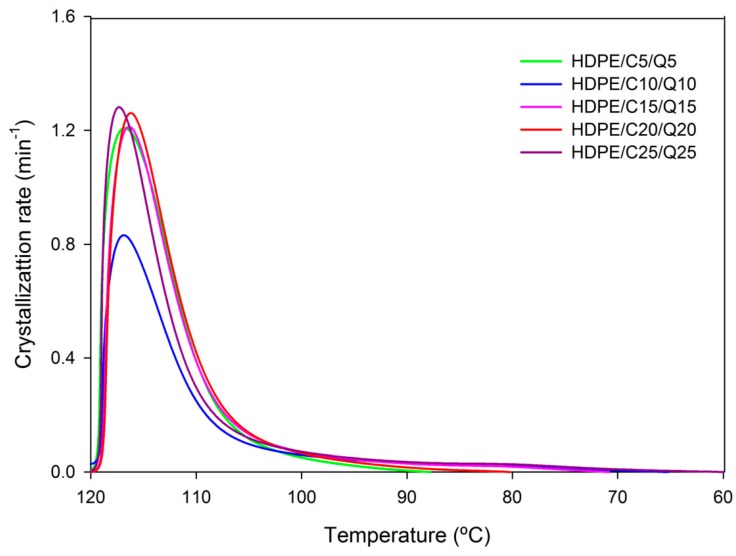
Crystallization rate versus temperature of HDPE, PE-*g*-MA, HDPE/C, HDPE/Q and HDPE/C/Q compounds.

Figure 3 shows plots of crystallization rate versus temperature for HDPE, PE-*g*-MA and HDPE compounds. The crystallization rate of HDPE/PE-g-MA blends ranged between 1 and 2 min^−1^ depending on the compatibilizer (PE-*g*-MA) content. The chitosan content subtly affected the crystallization rate, with HDPE/Q compounds presenting a narrow crystallization rate range, i.e., difference of 0.2 min^−1^ regardless the chitosan content, as further on commented even low, the most prominent chitosan effect was verified on the degree of crystallinity. Nevertheless, the compatibilizer slightly decreased the compounds crystallization rate, from ~1.5 min^−1^ compounds without PE-*g*-MA to ~1.3 min^−1^ compounds with PE-*g*-MA. For all compositions the crystallization peak was asymmetric. The crystallization was finished between 60 °C and 120 °C, however, 90% of crystallizable mass changes during the first 20 °C, the 10% remainder changes during the last 40 °C.

Table S2 of the Supplementary Materials presents crystallization parameters evaluated during melt crystallization. It is verified that the crystallization temperature of compounds did not change regardless of the compatibilizer and chitosan contents, it is 117 ± 0.5 °C, similar findings were obtained by Husseinsyah et al. [43] for polypropylene (PP)/chitosan systems. It was also observed that the degree of crystallinity (X_c_) was not modified upon addition of PE-*g*-MA and chitosan, measured values were 68% ± 10% agreeing with X_c_ data computed from X-ray diffraction. Even at high chitosan concentrations, the HDPE crystalline structure was maintained, and compounds had similar crystallization parameters, so plastic products obtained with HDPE/C/Q should have similar microstructures with bactericidal properties (transferred by chitosan as further on presented in the antibacterial properties) at relatively lower prices since chitosan is a low cost filler abundantly found.

**Figure 4:**
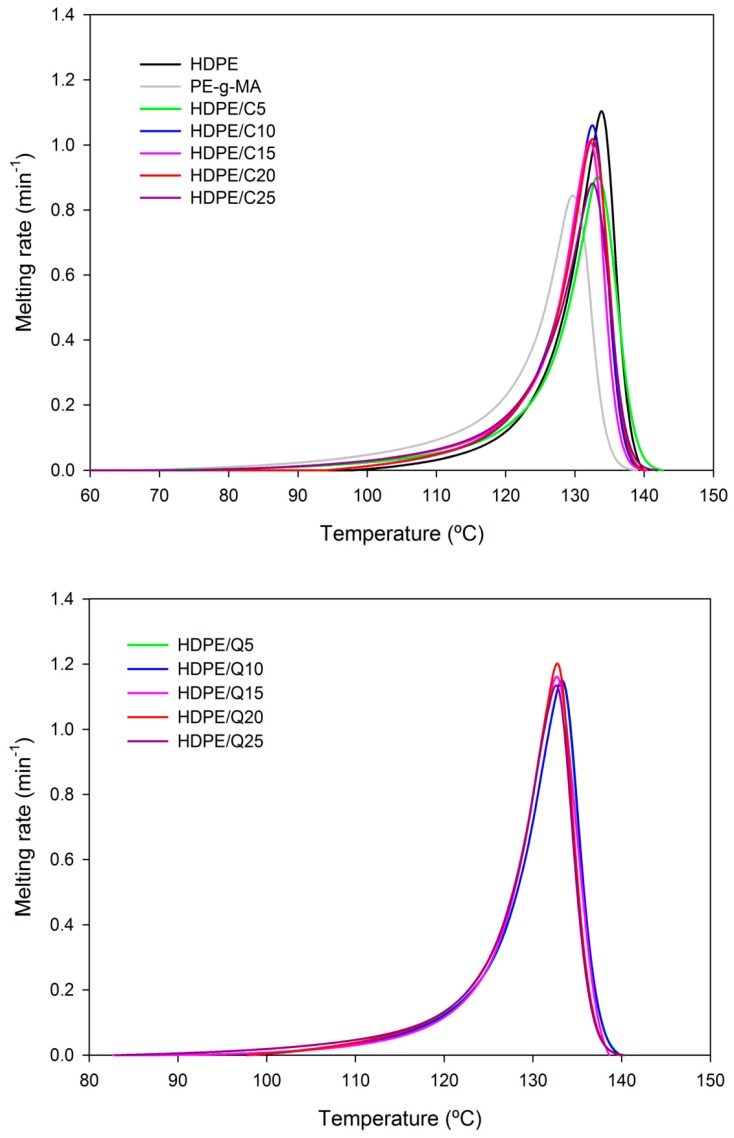

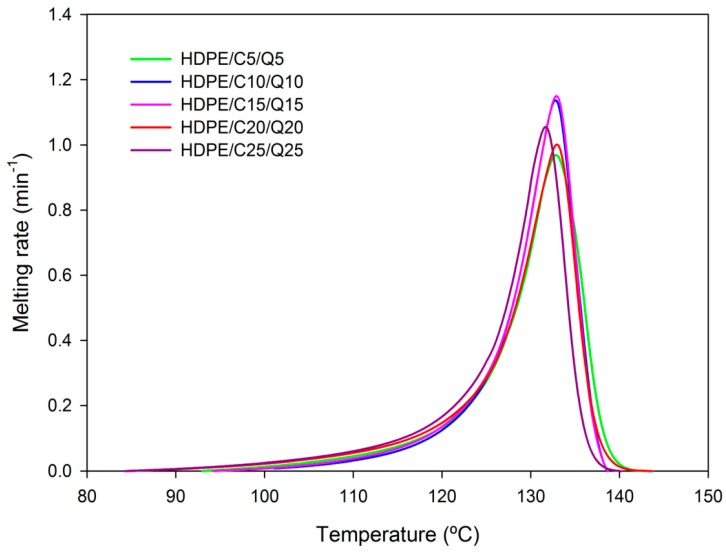
Melting rate versus temperature of HDPE, PE-g-MA, HDPE/C, HDPE/Q and HDPE/C/Q compounds.

Melting rates measured during the second heating are presented in Figure 4. As can be observed all compositions have similar melting behavior, with the melting peak temperature ~133 °C, neither PE-*g*-MA nor chitosan modified significantly HDPE melting parameter. The maximum melting rates cmax were around 0.8–1.2 min^−1^. Table S3 of the Supplementary Material shows the melting parameters of HDPE/C/Q compounds, which were like those of neat HDPE, meaning that HDPE/C/Q products might be processed using similar processing parameters, additionally taking advantage of differential properties transferred by PE-*g*-MA and chitosan as an example antibacterial character. It is also possible that HDPE/C/Q products have a biodegradable character and/or faster degradation rate, once chitosan is a natural polyaminosaccharide with biodegradability activity as inherent characteristic; it is worth mentioning chitosan is obtained at a relatively low cost and it is eco-friendly, safe for humans and for the environment.

**Figure 5:**
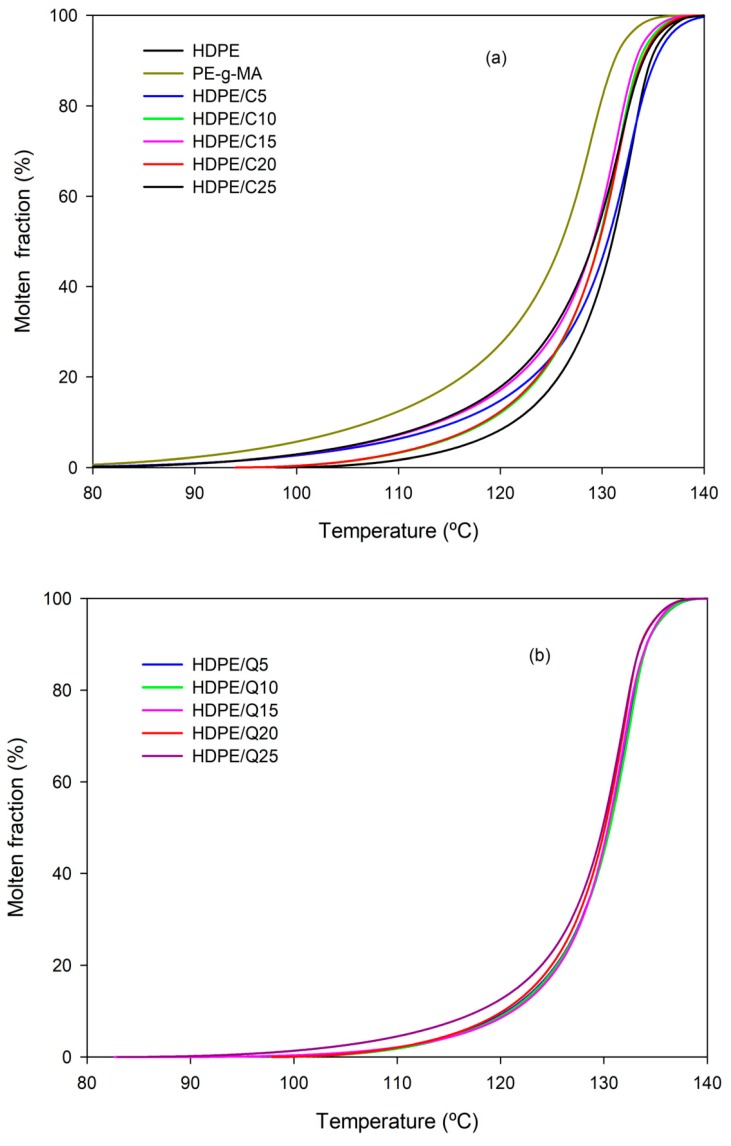

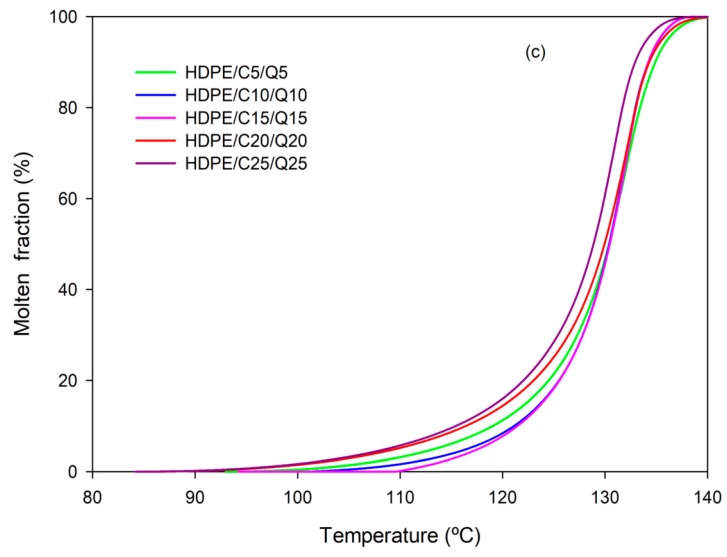
Molten fraction as a function of the temperature: (**a**) HDPE, PE-g-MA and HDPE/C compounds; (**b**) HDPE/Q compounds and (**c**) HDPE/C/Q compounds.

Figure 5 presents plots of the molten fraction as a function of the temperature for HDPE, HDPE/C and HDPE/C/Q. Independently the compatibilizer and chitosan contents, all experimented compounds exhibited almost the same melting behavior, the sigmoids shown in Figure 5 overlapped. These results support the previous findings made on the use of the same process parameters for neat HDPE, HDPE/C and HDPE/C/Q compounds.

**Figure 6:**
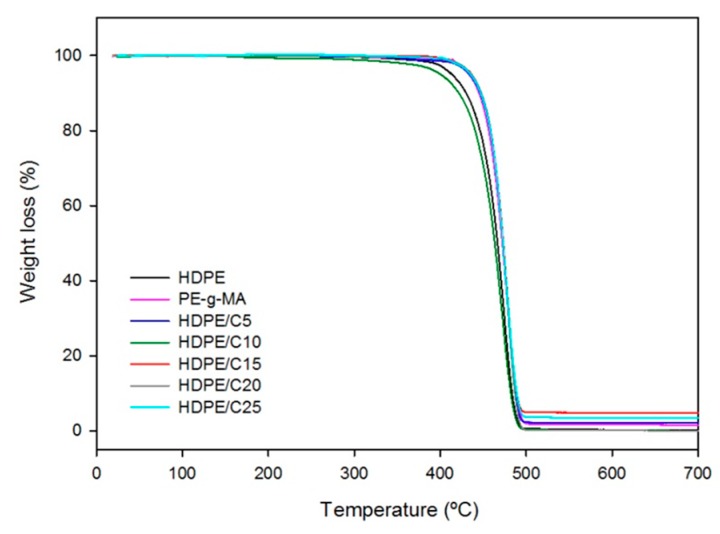
Thermogravimetry (TG) plots of HDPE, PE-g-MA and HDPE/C compounds.

Figure 6 shows thermogravimetry (TG) plots of HDPE, PE-g-MA and HDPE/C with 5%, 10%, 15%, 20% and 25% of compatibilizer (TGs of chitosan and HDPE are found in the Supplementary Material). Compounds with PE-*g*-MA have the weight loss like neat HDPE, in a single event that started near 300 °C and have the maximum degradation rate, T_½_ ~470 °C, the total weight loss was 97.7% ± 1.7%. Table 2 presents TG parameters for HDPE/C compounds, clearly the results indicate an increase in the thermal stability of HDPE/C compounds with increasing of PE-*g*-MA content, for neat HDPE decomposition it started at 300 °C, for HDPE/C25 it started at 352 °C, it is a good indicator since compounds should support higher temperatures without decomposition taking place.

**Figure 7:**
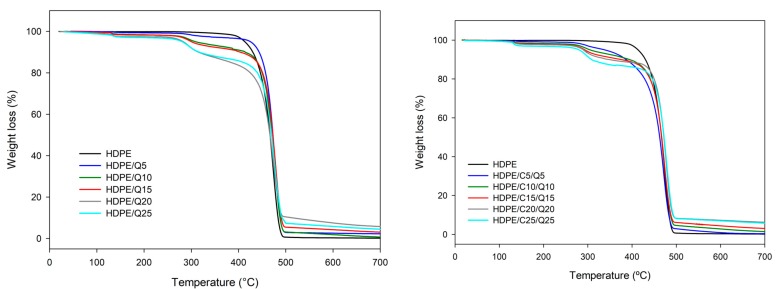
TG plots of HDPE, HDPE/Q (**left**) and HDPE/C/Q compounds (**right**).

Figure 7 shows TG plots for HDPE/Q and HDPE/C/Q compounds. They presented weight loss in three steps; the first took place between 120–150 °C and was related to 0.7–1.9% of weight loss, it suggests the initial humidity is associated with chitosan; the second step was visualized between 224–355 °C the weight loss corresponding to chitosan content, it was concerned to deacetylation and degradation of chitosan. The third step occurred between 332–515 °C due to HDPE degradation, macromolecular ruptures and the formation of carbonaceous residues originated from chitosan decomposition. Mir et al. [22] observed similar results in their researches with HDPE/chitosan.

The onset decomposition temperatures of compounds were lower than those of neat HDPE, meaning the maximum processing temperature for the compounds was limited by the begging of chitosan decomposition, i.e., the processing temperature should be lower than 200 °C, but it was high enough to process HDPE products. Table 3 and Table 4 show characteristic parameters for HDPE/Q and HDPE/C/Q compounds measured from TG plots.

According to Table 3 and Table 4 the average decomposition temperatures for the second and third stages of compounds were approximately 294.7 ± 4.3 °C and 470.8 ± 2.8 °C, respectively, regardless of the composition.

**Figure 8:**
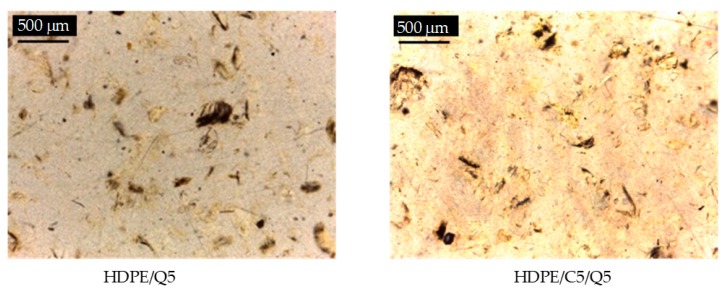

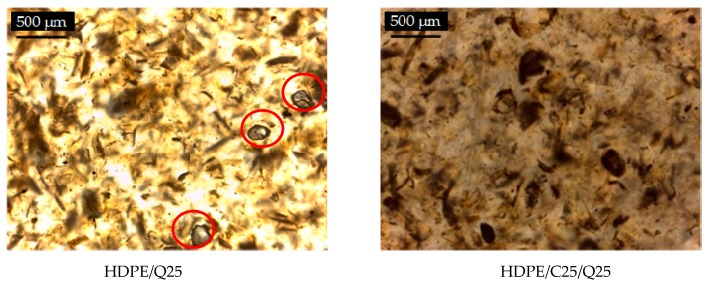
Optical microscopy images of HDPE/Q and HDPE/Q/C prepared with different quantities of Q and C.

A detailed analysis of HDPE morphology and its compounds was performed by optical microscopy (OM) and scanning electron microscopy (SEM). OM images of HDPE/C and HDPE/C/Q are illustrated in Figure 8. In the Supplementary Material, readers can find the OM image of the neat HDPE.

Figure 8 shows images for uncompatibilized compounds (HDPE/Q5 and HDPE/Q25); agglomerations of assorted sizes (dark regions) of chitosan particles were observed as also reported by Sunilkumar et al. [28] for low density polyethylene (LDPE)/chitosan. It was also verified voids (red circled), which were more evident for compounds with 25% chitosan (HDPE/Q25), that may be linked to the higher content of the filling (probably due to the hydrophobic character of chitosan) resulted in lower mechanical properties as further on presented.

Concerning the compatibilized compounds (HDPE/C5/Q5 and HDPE/C5/Q5), it was observed that chitosan was well dispersed, regardless of the content, it also showed a more uniform surface when compared with the uncompatibilized systems (HDPE/Q5 and HDPE/Q25). The compatibilizer PE-g-MA might provide a better interfacial adhesion between HDPE and chitosan that might have favored good dispersion, conducting to higher mechanical properties.

**Figure 9:**
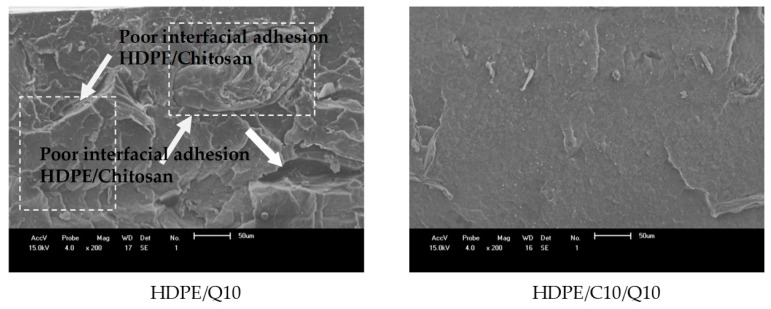

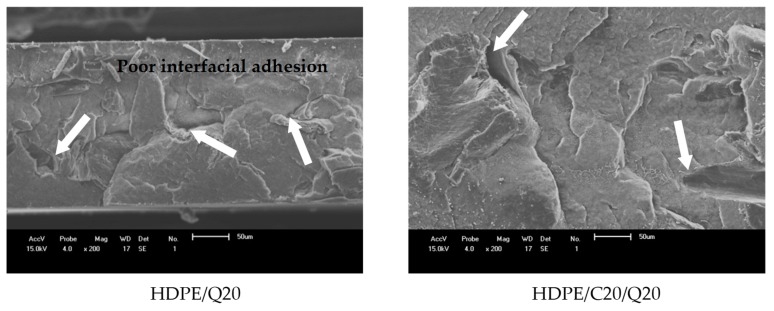
SEM images of HDPE/Q and HDPE/Q/C compounds.

SEM images of fractured surfaces for HDPE/Q and HDPE/Q/C compounds are presented in Figure 9, in the Supplementary Material readers can find SEM of HDPE, which presents a surface free from voids, with evidence of elastic and plastic deformation that might contribute to higher levels of elongation at break as shown in the mechanical properties section.

For uncompatibilized compounds (HDPE/Q10 and HDPE/Q20; Figure 9), a rough surface that was more intense at a higher content of chitosan was observed, i.e., HDPE/Q20 and HDPE/Q25 (Supplementary Material). Poor adhesion between HDPE and chitosan was clearly verified, as identified by the presence of voids, reflecting in lower mechanical properties.

According to Figure 9 the addition of PE-g-MA resulted in more homogeneous fractured surfaces (HDPE/C10/Q10 and HDPE/C10/Q10), layers of HDPE covering particles of chitosan and giving signs of a better interfacial interaction between the HDPE matrix and filling was also observed, it was evidenced by the break of particles (in fact a small agglomeration) instead of pulling-out them. However, even with PE-*g*-MA addition it was observed for some voids (dark regions), it intensified in HDPE/C20/Q20 and HDPE/C25/Q25 compounds (Supplementary Material). Similar results were observed by Husseinsyah et al. [43] when researching PP/chitosan treated with acrylic acid.

Compounds with chitosan showed smooth areas (as indicated by white arrows) characteristics of brittle fracture, while compatibilized compounds presented ridges and zones of elastic/plastic deformation suggesting a fracture with energy absorption (plastic behavior), this trend was most evident for the compatibilized compounds with lower chitosan concentrations, HDPE/C5/Q5 (Supplementary Material) and HDPE/C10/Q10. Nevertheless, in applications where high elongation is not a necessary, characteristic for HDPE/C/Q compounds, they may meet the other requirements, i.e., same crystalline structure, processing parameters and thermal stability to those of neat HDPE. Besides containing the antibacterial properties and low cost advantages supported by the chitosan addition, these requirements were satisfied in compounds even with high chitosan content.

**Figure 10:**
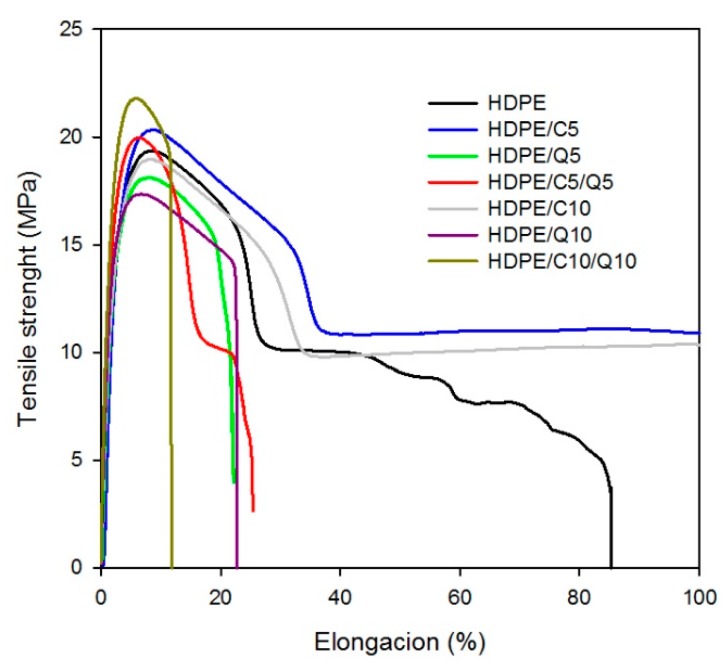
Tension versus deformation for HDPE, HDPE/C and HDPE/Q/C compounds.

Regarding the results for mechanical properties, Figure 10 presents tension versus deformation plots for HDPE, HDPE/Q, HDPE/C and HDPE/Q/C compounds. HDPE and HDPE/C present an extensive deformation before fracturing characteristic of highly elastic thermoplastics. Upon chitosan addition, the level of deformation abruptly decreased and material behaved as a common brittle plastic, behavior that agrees with the SEM images of HDPE/Q compounds where smooth zones were observable due to fracture without (Table 5) energy consuming. In general, PE-*g*-MA provided a subtle higher elastic modulus and wider deformation for HDPE compounds; it also contributed with a better dispersion of chitosan particles into HDPE matrix as shown in the MO and SEM images.

Table 5 presents results for elastic modulus (EM), tensile strength (TS) and elongation at break (EB) for neat HDPE and its compounds with chitosan and PE-*g*-MA. In general, the addition of chitosan resulted in higher EM, for HDPE/Q10 there was an increase of 18%, such behavior might be attributed to the movement restriction of HDPE macromolecular chains in front of chitosan particles. Regarding EB values, it decreased by 75% and 86% upon the addition of 5% and 10% of chitosan, similar behavior was obtained by Husseinsyah et al. [43]. Concerning the TS results neat HDPE and compounds with PE-*g*-MA and chitosan presented similar values, which were around 20 MPa. In summary, although there was a decrease in the elongation at break upon chitosan addition, the elastic modulus was only slightly modified and the tensile strength was unchanged. Therefore, in this work successful HDPE/C/Q compounds, with a compatibilizer and filler content up to 25 wt.% were prepared with the processing parameters similar to those used for neat HDPE, but with antibacterial as further on presented as well as with a low cost filling.

Table 6 presents impact strength (IS) results for neat HDPE and its compounds with PE-*g*-MA and chitosan. As shown upon the addition of PE-*g*-MA (5 and 10 wt.%) IS presented the same values as those found for neat HDPE agreeing with mechanical properties in tension previously reported, as well as, with SEM images. Upon chitosan addition there was a decrease on IS values, which was more intense for compounds with 10 wt.% of chitosan, in these compounds the energy absorption mechanism did not work properly, it is plausible to assume that chitosan particles behave as tension concentrators, once the crack reaches these particles it quickly propagates conducting a fracture with low energy consumption. As reported by Quiroz-Castillo et al. [25], since chitosan is a brittle material, an increase in chitosan content should result in a decrease in ductility. Lastly, upon chitosan and the PE-*g*-MA compatibilizer addition there was a wide decrease in IS of HDPE/C/Q compounds. This outcome might be due to the stiffening effect of chitosan and decreased deformability of a rigid interface between chitosan and the HDPE matrix, owing to the improved interfacial adhesion between the filler and matrix, which resulted in compounds more brittle than the uncompatibilized (HDPE/Q), at a similar filler loading. The same behavior was observed by Husseinsyah et al. [43] in a similar study.

**Figure 11:**
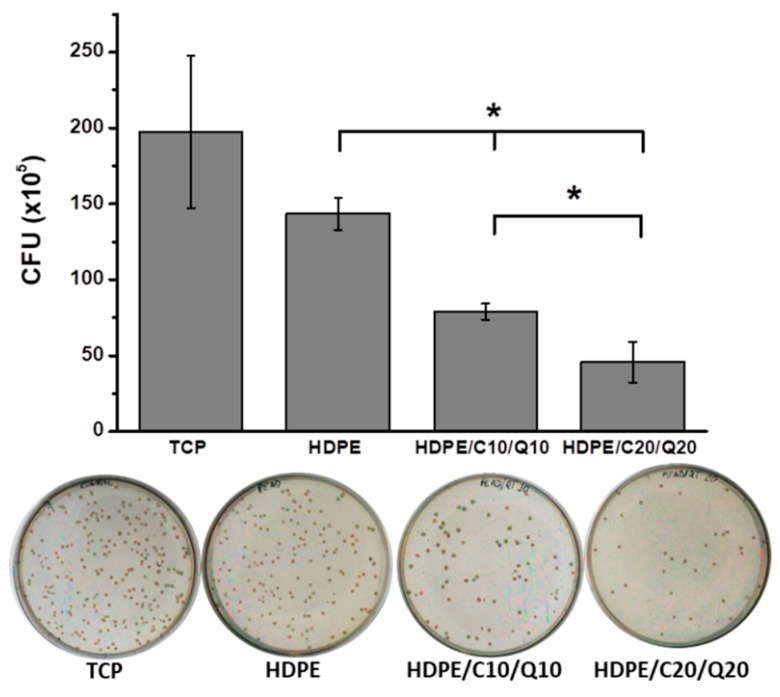
Colony-forming units (CFU) counting for HDPE and selected HDPE compounds relative to the tissue culture plastic (TCP) that was used as a control.

Selected compounds were tested for antibacterial properties. Figure 11 shows the significant effect of the modification on HDPE compounds reducing the number of viable CFU after their incubation for 12 h with RCV values of 51% and 31% for HDPE/C10/Q10 and HDPE/C20/Q20 respectively.

**Figure 12:**
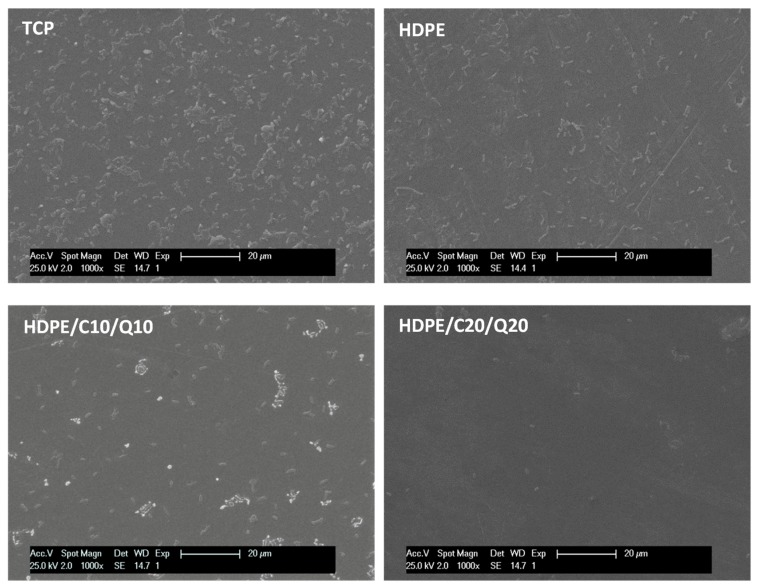
SEM observation of adhered *Escherichia coli* after incubation for 12 h onto tissue culture plastic (TCP), HDPE and selected HDPE compounds surfaces.

The reduction on bacterial adhesion observed for HDPE in comparison with tissue culture plastic (TCP) were attributable to the different chemical composition of both surfaces that affect free surface energy and thus cell adhesion. However, the significant effect observed between modified HDPE based surfaces (*p* > 0.5) were attributable to the bacteriostatic effect of chitosan that prevents bacteria to colonize and form biofilm on modified composites as illustrated in Figure 12.

## 4. Conclusions

HDPE compounds filled with chitosan and compatibilized with PE-g-MA were investigated in this work. Although, the compatibilizer and filler content ranged up to 25 wt.%, the HDPE crystalline structure and degree of crystallinity did not significantly change as verified by X-ray diffractograms. According to DSC thermograms, crystallization and melting parameters of compounds were like those of neat HDPE, suggesting HDPE/C/Q products might be similarly processed to neat HDPE and using the same machinery. PE-*g*-MA provided better dispersion of chitosan particles and improved the mechanical performance of compatibilized compounds. HDPE/C/Q compounds are lower cost and additionally have a bacteriostatic effect due to chitosan that prevent bacteria to colonize providing a wider range of applications than those commonly found for neat HDPE.

## Figures and Tables

**Table 1:** Component quantities and codes.

Code	Matrix (wt.%)		Chitosan (phr)
	HDPE	PE-*g*-MA	
HDPE	100	0	0
HDPE/C5	95	5	0
HDPE/C10	90	10	0
HDPE/C15	85	15	0
HDPE/C20	80	20	0
HDPE/C25	75	25	0
HDPE/Q5	95	0	5
HDPE/Q10	90	0	10
HDPE/Q15	85	0	15
HDPE/Q20	80	0	20
HDPE/Q25	75	0	25
HDPE/C5/Q5	95	5	5
HDPE/C10/Q10	90	10	10
HDPE/C15/Q15	85	15	15
HDPE/C20/Q20	80	20	20
HDPE/C25/Q25	75	25	25

**Table 2:** TG parameters of HDPE, PE-g-MA and HDPE/C.

Sample	T_o_ (°C)	T_e_ (°C)	T_½_ (°C)	ΔM (%)	Residue at 700 °C
HDPE	300.1	500.1	465.0	99.0	0.2
PE-*g*-MA (C)	315.4	505.6	472.3	98.5	1.6
HDPE/C5	320.2	506.5	470.2	97.3	2.2
HDPE/C10	290.2	503.4	463.5	99.2	0.2
HDPE/C15	367.4	507.2	471.3	95.0	4.7
HDPE/C20	345.1	508.4	472.1	99.4	0.1
HDPE/C25	352.5	509.6	471.3	95.8	3.5

T_o_: Onset temperature of weight loss. T_e_: End temperature of weight loss. T_½_: Temperature of maximum degradation rate. ΔM: Weight loss.

**Table 3:** Weight loss percentages evaluated from TG plots for HDPE/Q compounds.

Sample	Stages	T_o_ (°C)	T_e_ (°C)	T_½_ (°C)	ΔM (%)	Residue at 700 °C
HDPE/Q5	I	120.5	147.7	140.0	0.7	-
HDPE/Q10	I	120.9	150.4	133.3	1.1	-
HDPE/Q15	I	120.6	150.9	133.6	1.2	-
HDPE/Q20	I	120.5	146.3	134.5	1.5	-
HDPE/Q25	I	120.4	148.2	132.3	1.9	-
HDPE/Q5	II	255.9	346.3	303.3	1.8	-
HDPE/Q10	II	247.1	355.5	299.8	4.0	-
HDPE/Q15	II	245.5	340.1	296.9	4.9	-
HDPE/Q20	II	224.1	335.1	290.3	7.4	-
HDPE/Q25	II	246.8	331.9	291.8	6.7	-
HDPE/Q5	III	346.3	514.6	472.9	94.3	2.3
HDPE/Q10	III	355.5	503.9	472.5	86.2	0.7
HDPE/Q15	III	340.1	504.1	473.3	83.8	3.1
HDPE/Q20	III	335.1	504.7	470.5	72.0	5.8
HDPE/Q25	III	331.9	509.1	473.8	77.0	3.1

**Table 4:** Weight loss percentages evaluated from TG plots for HDPE/Q/C compounds.

Sample	Stages	T_o_ (°C)	T_e_ (°C)	T_½_ (°C)	ΔM (%)	Residue at 700 °C
HDPE/C5/Q5	I	114.4	165.7	141.5	0.5	-
HDPE/C10/Q10	I	112.5	170.7	133.3	0.9	-
HDPE/C15/Q15	I	118.2	155.2	134.5	1.6	-
HDPE/C20/Q20	I	120.4	166.4	136.5	1.8	-
HDPE/C25/Q25	I	119.1	145.3	126.7	1.5	-
HDPE/C5/Q5	II	245.8	350.2	295.6	2.9	-
HDPE/C10/Q10	II	218.3	364.5	291.4	3.2	-
HDPE/C15/Q15	II	237.7	328.5	292.9	4.8	-
HDPE/C20/Q20	II	235.3	320.2	294.1	6.2	-
HDPE/C25/Q25	II	254.3	366.1	290.5	7.0	-
HDPE/C5/Q5	III	350.2	502.2	465.6	88.8	0.4
HDPE/C10/Q10	III	364.1	507.3	467.2	84.1	1.5
HDPE/C15/Q15	III	328.5	506.3	468.8	81.2	3.1
HDPE/C20/Q20	III	320.3	506.7	470.7	78.7	6.3
HDPE/C25/Q25	III	366.2	500.1	473.2	75.1	6.0

**Table 5:** Mechanical properties of HDPE, HDPE/C, HDPE/Q and HDPE/Q/C compounds.

Sample	EM (MPa)	TS (MPa)	EB (%)
HDPE	1046 ± 17	19.5 ± 0.3	117.8 ± 31
HDPE/C5	1068 ± 41	20.0 ± 0.3	Not registered
HDPE/C10	1047 ± 47	19.5 ± 0.3	Not registered
HDPE/Q5	1084 ± 57	18.2 ± 0.5	28.9 ± 3.9
HDPE/Q10	1238 ± 94	19.5 ± 0.5	16.1 ± 5.1
HDPE/C5/Q5	1412 ± 109	20.8 ± 0.6	18.9 ± 3.6
HDPE/C10/Q10	1499 ± 94	21.6 ± 0.2	11.6 ± 1.6

**Table 6:** Impact strength of HDPE, HDPE/C, HDPE/Q and HDPE/C/Q.

Sample	IS (J/m)
HDPE	63 ± 2.8
HDPE/C5	58 ± 6.6
HDPE/C10	59 ± 3.2
HDPE/Q5	55 ± 4.4
HDPE/Q10	50 ± 2.0
HDPE/C5/Q5	39 ± 3.3
HDPE/C10/Q10	41 ± 2.5

## Supplementary Materials

The following are available online at https://www.mdpi.com/2073-4360/11/10/1559/s1.
