# Supplementary material for: HDPE/Chitosan Composites Modified with PE-g-MA. Thermal, Morphological and Antibacterial Analysis
Source: Polymers (Basel). 2019 Sep 25;11(10):1559. doi: 10.3390/polym11101559 (PMC6835920; doi:10.3390/polym11101559)
Supplement: Supplementary file 1 [file polymers-11-01559-s001.pdf]

Supplementary Material

X Ray Diffraction (XRD)

Table S1. Degree of crystallinity (Xc) for neat HDPE, PE-g-MA, chitosan, HDPE/C, HDPE/Q and HDPE/C/Q compounds, measured from X ray diffractograms and applying Eq. 1

Compound	X_c (%)
HDPE	57.1
PE-g-MA	54.8
Chitosan	24.2
HDPE/C5	56.7
HDPE/C10	60.2
HDPE/C15	65.1
HDPE/C20	68.0
HDPE/C25	78.7
HDPE/Q5	63.5
HDPE/Q10	62.3
HDPE/Q15	66.2
HDPE/Q20	68.1
HDPE/Q25	68.0
HDPE/C5/Q5	61.0
HDPE/C10/Q10	57.7
HDPE/C15/Q15	61.7
HDPE/C20/Q20	63.3
HDPE/C25/Q25	66.7

Differential Scanning Calorimetry (DSC)

Table S2. Crystallization parameters evaluated from DSC scans during cooling for HDPE, PE-g-MA, HDPE/C, HDPE/Q and HDPE/C/Q.

Sample	$\Delta T_{c\ (1\text{-}99\%)}$ (°C)	T_c (°C)	ΔH_c (J/g)	X_c (%)	c_{max} (min^{-1})	$\Delta T_{½}$ (°C)	$\tau_{½}$ (min)
HDPE	119.4-102.9	116.9	168.8	57.6	1.4	6.8	0.54
PE-g-MA	116.9-60.8	76.2	186.1	63.5	1.1	7.4	0.73
HDPE/C5	118.9-69.2	116.0	215.0	73.4	1.1	7.9	0.65
HDPE/C10	120.3-105.0	118.0	207.3	70.8	1.4	6.3	0.52
HDPE/C15	118.9-77.8	116.6	206.2	70.4	1.3	6.1	0.56
HDPE/C20	119.7-102.7	117.7	168.2	57.4	13	6.1	0.54
HDPE/C25	119.8-74.5	117.0	170.2	58.0	1.0	8.3	0.68
HDPE/Q5	118.8-102.8	116.3	178.2	60.8	1.5	6.1	0.54
HDPE/Q10	119.2-103.0	116.9	174.5	59.5	1.5	6.1	0.51
HDPE/Q15	119.5-76.9	116.8	216.0	73.7	1.3	6.4	0.87
HDPE/Q20	119.2-103.0	117.0	187.5	64.0	1.5	5.6	0.50
HDPE/Q25	119.4-80.7	117.5	228.6	78.0	1.3	5.9	0.6
HDPE/C5/Q5	119.5-96.3	116.8	190.7	65.1	1.2	7.3	0.67
HDPE/C10/Q10	119.2-79.0	116.9	204.0	69.6	1.2	6.5	0.57
HDPE/C15/Q15	119.1-74.2	116.4	208.6	71.2	1.2	6.5	0.79
HDPE/C20/Q20	119.0-83.9	116.2	189.1	64.5	1.3	6.7	0.67
HDPE/C25/Q25	119.3-65.1	117.3	225.5	77.0	1.3	5.9	0.68

ΔT_c= crystallization interval; T_c = crystallization peak temperature; ΔH_c = crystallization enthalpy; X_c = degree of crystallinity; c_{max}. = maximum crystallization rate; $\Delta T_{½}$ = width of crystallization peak at half weight; $\tau_{½}$= time to reach 50% of crystallization.

Table S3. Melting parameters evaluated from DSC scans during the second heating for HDPE, PE-g-MA, HDPE/C, HDPE/Q and HDPE/C/Q.

Sample	$\Delta T_{m\,(1\text{-}99\%)}$ (°C)	T_m (°C)	ΔH_m (J/g)	X_c (%)	$c_{máx}$ (min^{-1})	$\Delta T_{½}$ (°C)	$\tau_{½}$ (min)
HDPE	107.6-137.8	133.9	177.8	60.7	1.1	7.0	3.1
PE-g-MA	85.2-134.7	129.7	187.3	64.0	0.8	8.2	5.5
HDPE /C5	91.1-140.0	133.6	202.4	69.1	1.0	8.2	5.5
HDPE /C10	106.5-136.8	132.5	222.9	76.1	1.1	7.0	3.5
HDPE /C15	91.4-136.5	132.3	209.4	71.5	1.0	6.9	5.0
HDPE /C20	103.5-137.1	132.5	182.4	62.3	1.0	7.4	3.4
HDPE /C25	91.3-137.7	132.7	154.3	52.7	*0.9*	8.2	5.0
HDPE /Q5	107.0-137.4	133.3	191.4	65.3	1.1	6.5	3.1
HDPE /Q10	107.1-137.3	133.3	187.2	63.9	1.2	6.7	3.1
HDPE /Q15	105.2-136.9	132.7	188.5	64.3	1.2	6.7	4.9
HDPE /Q20	106.7-136.7	132.8	209.7	71.6	1.2	6.2	3.0
HDPE /Q25	98.0-136.7	132.6	228.3	77.9	1.1	6.4	4.8
HDPE /C5/Q5	96.5-138.3	132.8	202.5	69.1	0.97	7.9	3.9
HDPE /C10/Q10	102.2-138.0	132.8	172.0	58.7	1.14	6.9	5.6
HDPE /C15/Q15	109.9-138.0	132.9	178.7	61.0	1.15	6.8	8.3
HDPE /C20/Q20	89.1-140.1	133.0	200.2	68.3	1.0	7.1	4.6
HDPE /C25/Q25	88.6-138.1	131.7	217.0	74.1	1.1	6.7	4.57

ΔT_m= melting temperature interval; T_m = melting peak temperature; ΔH_m = melting enthalpy; X_c = degree of crystallinity; $c_{max.}$ = maximum rate of melting; $\Delta T_{½}$ = width of melting peak at half weight; $\tau_{½}$= time to reach 50% of melting.

Thermogravimetry (TG)

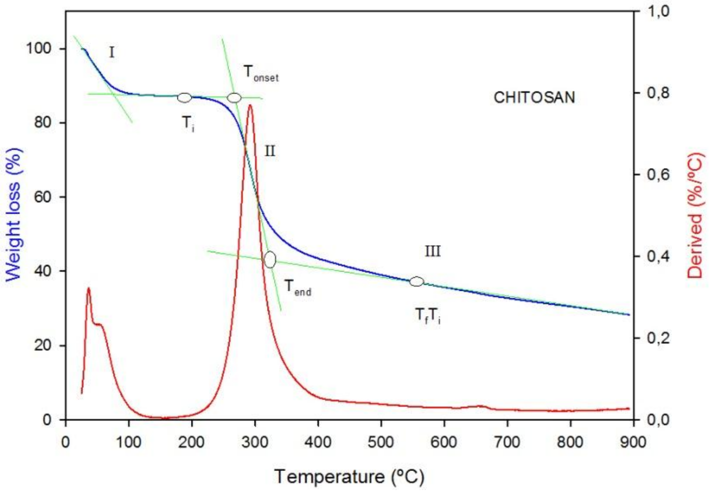

Figure S1. TG and DTG plots of chitosan.

Table S4. TG parameters of chitosan (Q).

Sample	Stages	T_o (°C)	T_e (°C)	$T_{1/2}$ (°C)	ΔM (%)	Residue at 700ºC
CHITOSAN (Q)	I	26.7	150.1	51.8	12.2	-
	II	200.1	560.3	295.0	43.6	-
	III	560.3	900.0	612.4	14.9	32.8

T_o = T_{onset}, T_e = T_{end}, $T_{1/2}$ = temperature at half of the event, ΔM= weight loss during the event

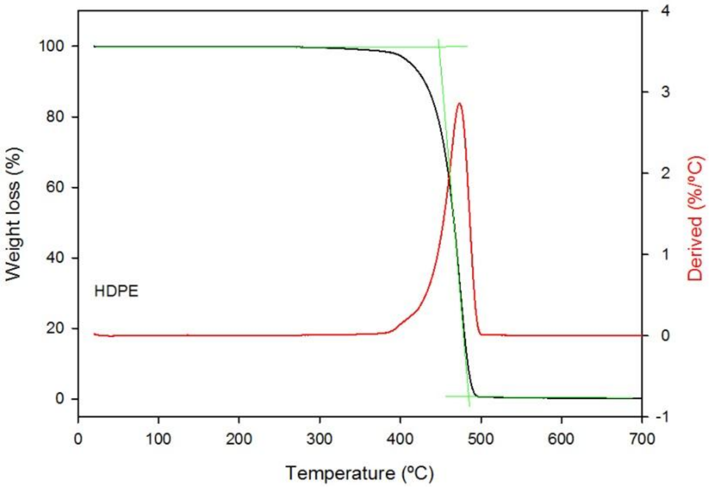

Figure S2. TG and DTG plots of HDPE.

Optical Microscopy (OM)

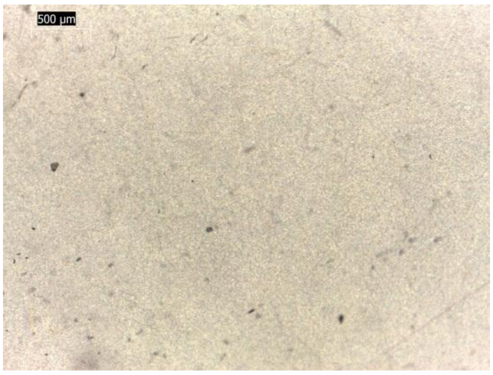

Figure S3. Optical microscopy image of HDPE.

Scanning Electron Microscopy (SEM)

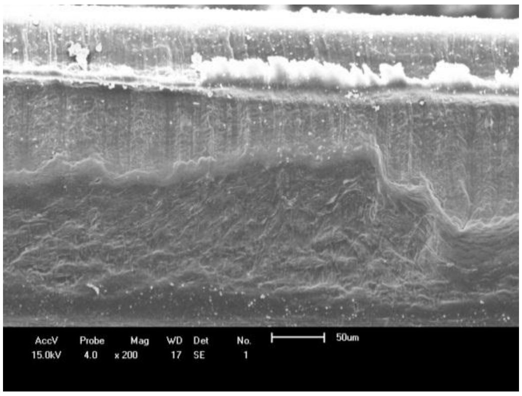

Figure S4. SEM image of HDPE.

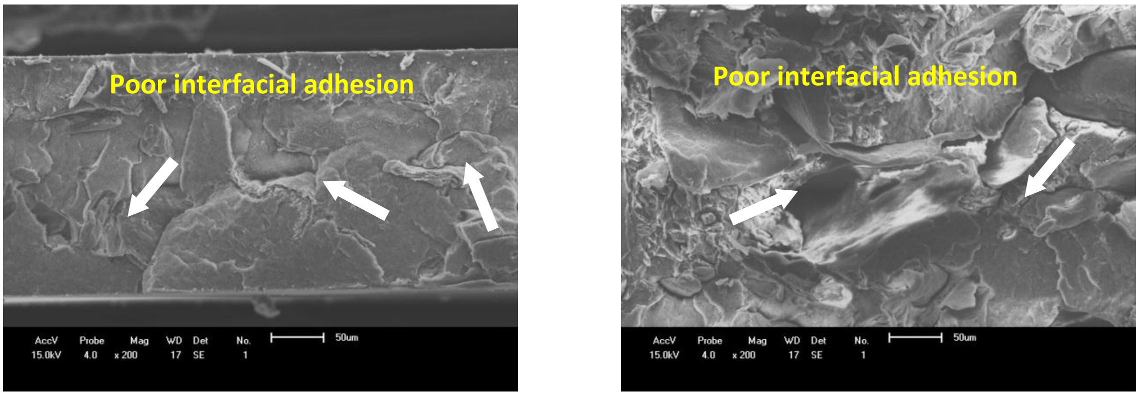

Figure S5. SEM images of HDPE/Q20 (left) and HDPE/Q25 (right).

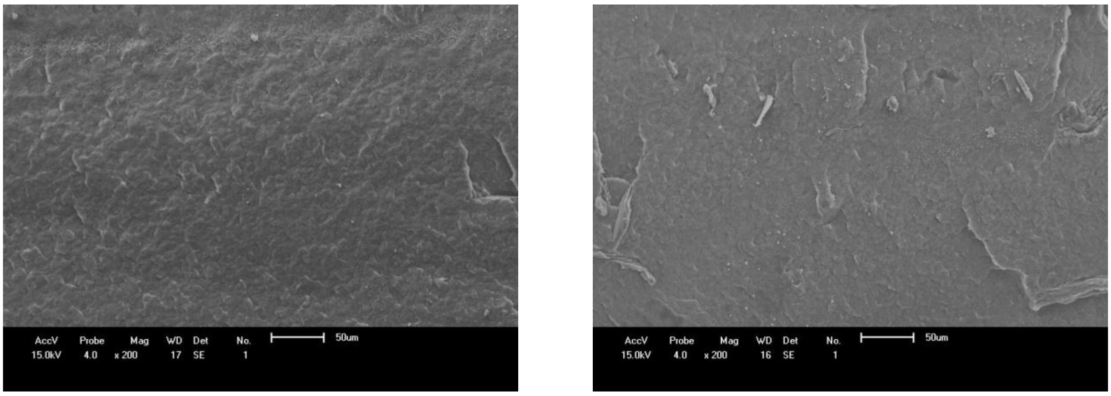

Figure S6. SEM images of HDPE/C5/Q5 (left) and HDPE/C10/Q10 (right).
